# Grocco's Paravertebral Triangle and Its Value

**Published:** 1909-07-24

**Authors:** 


### Medicine.

### GROCCO'S PARAVERTEBRAL TRIANGLE AND ITS VALUE.

Grocco's sign scarcely deserves the contemptuous neglect into which it has fallen, in many quarters, together with various recent mushroom growths of diagnostic technique, which, after hindering progress for a little while, pass each into oblivion. For there really seems something to be learned from it. Every physician knows the difficulty, in cases of pleuritic effusion, of determining whether the abnormal physical signs, which may persist for weeks after the chest has been tapped, are due to the persistence of fluid, or are only the after-effect of compression of the lung by fluid that has now quite gone. It is stated that, if there had been dulness in Grocco's paravertebral triangle during the early stages of the effusion, and if that dulness subsequently disappears, even though all the other signs remain much the same, the presumption is strongly in favour of the fluid having cleared up. If Grocco's sign can teach us nothing more than this it is a valuable point in diagnosis, and is well worthy of consideration.

### Grocco's Sign.

The sign consists in the occurrence in cases of pleuritic effusion and empyema of a triangular area of dulness at the lower part of the healthy side of the chest posteriorly. Th ebase of the triangle lies approximately along the eleventh rib in the horizontal plane. The apex is rather below the upper level of dulness on the diseased side. The sides of the triangle are formed by the vertical median line of the spine internally, and externally by an oblique line joining the apex and the outer extremity of the base. The base itself varies in length from an inch and a half to four inches, the exact length varying roughly with the size of the effusion upon the other side. This triangle is now generally known as Grocco's Paravertebral Triangle.

As regards the value of the sign, all physicians will agree that any aid to the diagnosis of effusion in doubtful cases is to be welcomed. Most observers who have studied the sign, moreover, insist upon its value. Dr. Moorhead recently read a paper upon it before the Section of Medicine of the Royal Academy of Medicine in Ireland, and his own conclusions,* stated briefly, are as follows:

(1). The sign is found only in cases of pleural effusion, and it is absent in pneumonia and in pulmonary fibrosis. Dr. Moorhead has been unable to satisfy himself of the presence of a bilateral paravertebral triangle of dulness, as described by Dr. Ewart in ascites. He has met no case of pericardial effusion with dorsal dulness since he began to study the sign, nor any case of subphrenic abscess; but he thinks the latter at least might cause a small paravertebral triangle of dulness like that of pleural effusion.

(2). The sign is not invariably present in pleural effusion. This is in agreement with the earlier observations of others, though some clinicians now insist that it always accompanies effusion. Some maintain that the dulness is best marked when the effusion is on the right side; but this is not by any means a universal rule.

(3). The sign, when present, is of great value; its absence is of much less import.

(4). If the sign has once been observed in a given case, its disappearance is of great value in distinguishing between thickened pleura on the one hand and persistent effusion on the other.

(5). Dr. Moorhead never observed the dulness to extend above the fourth dorsal spine even when the effusion filled the whole of the opposite side of the chest. This observation does not agree with the statements of Dr. Ewart and others; but he is convinced of its correctness in his own cases, and he believes this to be due to the fact that the easily movable "mediastinal mesentery," as he calls it, does not extend above the fourth dorsal spine.

* *Dublin Journal of Medical Science.* 1909, p. 419.

### Theories as to its Causation.

There are three alternative explanations of the cause of Grocco's sign:—

(1). Some observers hold that the dulness normally exists on both sides, but that it has hitherto been overlooked. They say it is due to the shape and increasing thickness from above downwards of the erectores spinæ muscles. This view is easily disproved by simple percussion of normal individuals.

(2). Others hold that the dulness is due to a damping effect exerted by the effusion, which prevents the vertebral column from conducting the resonance of the sound side. This damping effect, it is urged, would be exerted to a greater extent at the lower part of the thorax when the costo-vertebral space is deep and the fluid abundant, than above where the fluid is more scanty and the costo-vertebral space shallow.

(3). A more likely explanation is that which attributes the dulness to mediastinal displacements. An effusion upon the right side, for example, pushes all the movable contents of the mediastina towards the left, thereby causing more or less compression of the lower part of the left lung, with consequent impairment of note at the left base reaching a variable distance outward from the middle line. There is a "mesentery" of the mediastinum extending from the back of the pericardium to the vertebral column, not usually described by anatomists, but well seen in a transverse section of a chest hardened in formalin. This mediastinal mesentery extends upwards only to about the level of the fourth dorsal vertebra. In at least one case of extensive pleural effusion, in a still-born child, displacement of this mediastinal mesentery over to the sound side has been demonstrated. That this displacement may well be the cause of Grocco's paravertebral triangle of dulness is proved by the experimental injection of gelatine into one pleural cavity of a cadaver, the characteristic triangle of dulness appearing at the other base with displacement of the mediastinal mesentery towards the uninjected side.

